# Association of single nucleotide polymorphism rs6983267 with the risk of prostate cancer

**DOI:** 10.18632/oncotarget.8186

**Published:** 2016-03-18

**Authors:** Yuan Yang, Wenjing Wang, Liangcai Zhang, Shihua Zhang, Guiyou Liu, Yingcui Yu, Mingzhi Liao

**Affiliations:** ^1^ College of Life Sciences, Northwest A&F University, Yangling, Shaanxi, China; ^2^ School of Life Sciences, Fudan University, Shanghai, China; ^3^ Research Center for Translational Medicine, East Hospital, Tongji University School of Medicine, Shanghai, China; ^4^ Department of Statistics, Rice University, Houston, TX, USA; ^5^ Department of Biostatistics, School of Science, Anhui Agricultural University, Hefei, China; ^6^ Genome Analysis Laboratory, Tianjin Institute of Industrial Biotechnology, Chinese Academy of Sciences, Tianjin, China; ^7^ College of Natural Resources and Environment, Northwest A&F University, Yangling, Shaanxi, China

**Keywords:** prostate cancer, rs6983267, meta-analysis, risk, association

## Abstract

Many studies have investigated the association between single nucleotide polymorphism (SNP) rs6983267 and the risk of prostate cancer. However, results of these studies are inconsistent. Therefore, we summarised available data and performed a meta-analysis to determine this association. Relevant articles were identified by searching the PubMed, Web of Science and Embase database. Odds ratios (ORs) with 95% confidence intervals (CIs) were calculated using random effects model. We used dominant model (GG + TG vs TT), recessive model (GG vs TG + TT) and additive model (GG +TT vs TG) to determine the association between the rs6983267 polymorphism and risk of prostate cancer. Summary, 9 studies involving 8726 participants were included in this meta-analysis. Overall, though no association was observed between the rs6983267 polymorphism and risk of prostate cancer, subgroup analysis according to ethnicity showed a significant association between the rs6983267 polymorphism and risk of prostate cancer among white European men [recessive model: GG vs TG + TT, OR=1.21, (95% CI: 1.03, 1.42), P=0.02]. Our results indicate that the GG genotype of the rs6983267 polymorphism will increase individual susceptibility to prostate cancer in white European men.

## INTRODUCTION

Genetic epidemiology of prostate cancer is complex, and its incidence varies significantly among ethnic groups. This variation may be because of an association between genetic and environmental factors [1, 2]. However, the genetic epidemiology of prostate cancer is unclear.

Prostate cancer is the most frequently diagnosed cancer in many developing countries [3]. The incidence of prostate cancer was low in the past few decades. However, better living conditions and population aging have increased the incidence of prostate cancer rapidly. According to the WHO statistics, prostate cancer is the sixth leading cause of death due to cancer in men, with an estimated 258000 deaths in 2008 [4].

It's reported that age, ethnicity and familial history of prostate cancer increase the risk of prostate cancer [5]. Genome-wide association studies (GWASs) have shown that some single nucleotide polymorphisms (SNPs) are risk factors of prostate cancer [6, 7]. Therefore, it is important to elucidate the presence of these risk alleles in different populations [8]. Polymorphism rs6983267 which locates on chromosome 8q24 is a G/T single-nucleotide variation [9] and is associated with prostate cancer.

There are many studies which focus on the relationship between this SNP and Prostate Cancer. In 2007, Zheng *et al.* suggested that the rs6983267 polymorphism was associated with prostate cancer in European–American men [10]. In 2008, Fletcher *et al*. showed an association between the rs6983267 polymorphism and the risk of prostate cancer in English and Scottish men, and Cheng *et al.* confirmed this association in European–American men [11, 12]. In 2009, Miao Liu *et al.* observed this association in Japanese men [13]. In 2012, Joung *et al.*, Chan *et al.* and Ho *et al.* did not observe any significant association between this polymorphism and the risk of prostate cancer [14–16], which was consistent with the findings of Branković *et al.* and Oskina *et al.* in 2013 [17, 18]. These studies included Siberian, Serbian, Scottish, Korean, Chinese, Japanese, European Americans and English men. Besides this SNP, we also found that there are more SNPs associated with Prostate Cancer [19, 20]. Thought these researches are all based on experiment results, they always do not show consistent results and the roles rs6983267 plays in Prostate Cancer is unclear. Therefore, there is a need to make it clear whether this polymorphism is associated with Prostate Cancer.

In the present study, we conducted a meta-analysis based on previously published studies on the association between the rs6983267 polymorphism and risk of prostate cancer to clarify the impact of this polymorphism. As an article about meta-analysis on the disease genetic study, we focus on the relationship between single-nucleotide variation and Prostate Cancer. Based on the integrated analysis about previous researches, we did found SNP rs6983267 has significantly correlation with Prostate Cancer in European population. Considering the effects of meta-analysis, this finding should be the most robust and believable for the related research currently. As to the location and function about the related genes of SNP rs6983267, we will follow up the latest research progress and take deep research in future.

## RESULTS

### Literature search

**Figure 1:**
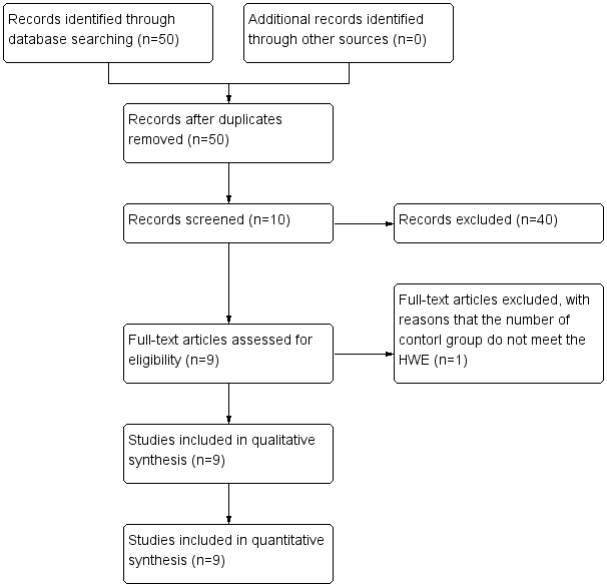
Flow diagram of the process for study selection

A flow diagram for the study selection process is shown in Figure 1. In all, 50 studies were identified using the search strategy. Of these, 40 studies were excluded because they did not have sufficient data and 10 studies were screened further. Of the 10 studies, 1 study was excluded because the genotype of the case-control group did not satisfy the Hardy-Weinberg Equilibrium (HWE). Finally, 9 studies were included in the meta-analysis.

### Study characteristics

The primary characteristics of the 9 studies are summarised in Table 1. In all, 8726 participants (5008 cases and 3718 controls) were included in the meta-analysis. The studies were divided into 2 groups based on the ethnicity of study participants, i.e., studies involving white European men (6 studies) and those involving men from East Asia countries (3 studies).

**Table 1:** Characteristics of the 9 studies included in the meta-analysis.

Author (Publication Date)	Country	Case			Control			Susceptibility^a^	P value of HWE^b^
		GG	TG	TT	GG	TG	TT		
Natalia A. Oskina (2013)	Siberian	114	186	89	87	177	77	N	0.471
Ana S. Branković (2013)	Serbian	53	80	17	25	49	26	N	0.842
CKM Ho (2012)	Scottish	42	104	70	46	136	66	N	0.102
Jae Y. Joung (2012)	Korean	46	92	56	31	86	51	N	0.618
Jason Yongsheng Chan (2012)	Chinese	63	136	89	23	74	47	N	0.493
Miao Liu (2009)	Japanese	25	151	147	59	181	151	Y	0.694
S. Lilly Zheng (2007)	European–American	495	771	285	142	299	132	Y	0.293
Olivia Fletcher (2008)	English and Scottish	408	734	338	371	653	312	Y	0.404
Iona Cheng (2008)	European–American	126	215	76	105	206	106	Y	0.807

a “Y” indicates an association between the rs6983267 polymorphism and risk of prostate cancer; N indicates no association between the rs6983267 polymorphism and risk of prostate cancer.

b HWE, Hardy–Weinberg equilibrium; P > 0.05 indicates that the participants in the control group met the HWE.

### Association between the rs6983267 polymorphism and risk of prostate cancer

**Figure 2:**
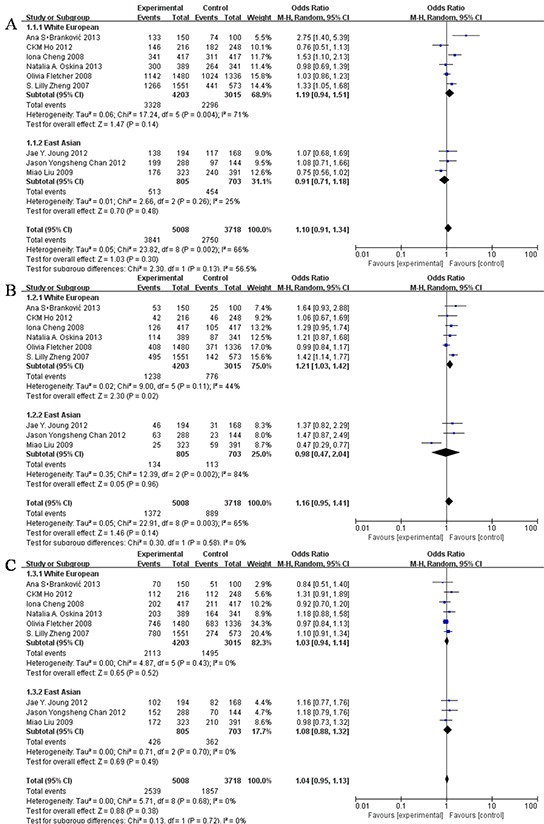
Forest Plot of different model **A.** Dominant model (GG + TG vs TT); **B.** Recessive model (GG vs TG + TT); **C.** Additive model (GG +TT vs TG).

Forest plot of overall and subgroup analyses with different models on the association between the rs6983267 polymorphism and risk of prostate cancer is shown in Figure 2.

Relative risk of the association between GG genotype of the rs6983267 polymorphism and risk of prostate cancer was analysed by performing overall and subgroup analyses. In dominant model [overall: OR = 1.10 (95% CI: 0.91, 1.34); test for overall effect, P = 0.30; heterogeneity, *I^2^* = 66%; white European men: OR = 1.19 (95% CI: 0.94, 1.51); test for overall effect, P = 0.14; heterogeneity, *I^2^*= 71%; East Asian men: OR = 0.91 (95% CI: 0.71, 1.18); test for overall effect, P = 0.48; heterogeneity, *I^2^* = 25%]. In recessive model [overall: OR = 1.16 (95% CI: 0.95, 1.41); test for overall effect, P = 0.14; heterogeneity, *I^2^*= 65%; white European men: OR = 1.21 (95% CI: 1.03, 1.42); test for overall effect, P = 0.02; heterogeneity, *I^2^*= 44%; East Asian men: OR = 0.98 (95% CI: 0.47, 2.04); test for overall effect, P = 0.96; heterogeneity, *I^2^* = 84%]. In additive model [overall: OR = 1.04 (95% CI: 0.95, 1.13); test for overall effect, P = 0.38; heterogeneity, *I^2^* = 0%; white European men: OR = 1.03 (95% CI: 0.94, 1.14); test for overall effect, P = 0.52; heterogeneity, *I^2^*= 0%; East Asian men: OR = 1.08 (95% CI: 0.88, 1.32); test for overall effect, P = 0.49; heterogeneity, *I^2^* = 0%].

Of the 9 studies, 4 reported an association between the rs6983267 polymorphism and risk of prostate cancer while 5 did not. The meta-analysis did not show any association between the rs6983267 polymorphism and risk of prostate cancer [recessive model: GG vs TG + TT, OR = 1.16 (90% CI: 0.98, 1.36), P = 0.14]. However, subgroup analysis showed a significant association between the rs6983267 polymorphism and risk of prostate cancer in white European men [recessive model: GG vs TG + TT, OR = 1.21 (90% CI: 1.06, 1.39), P = 0.02].

### Assessment of publication bias

**Figure 3:**
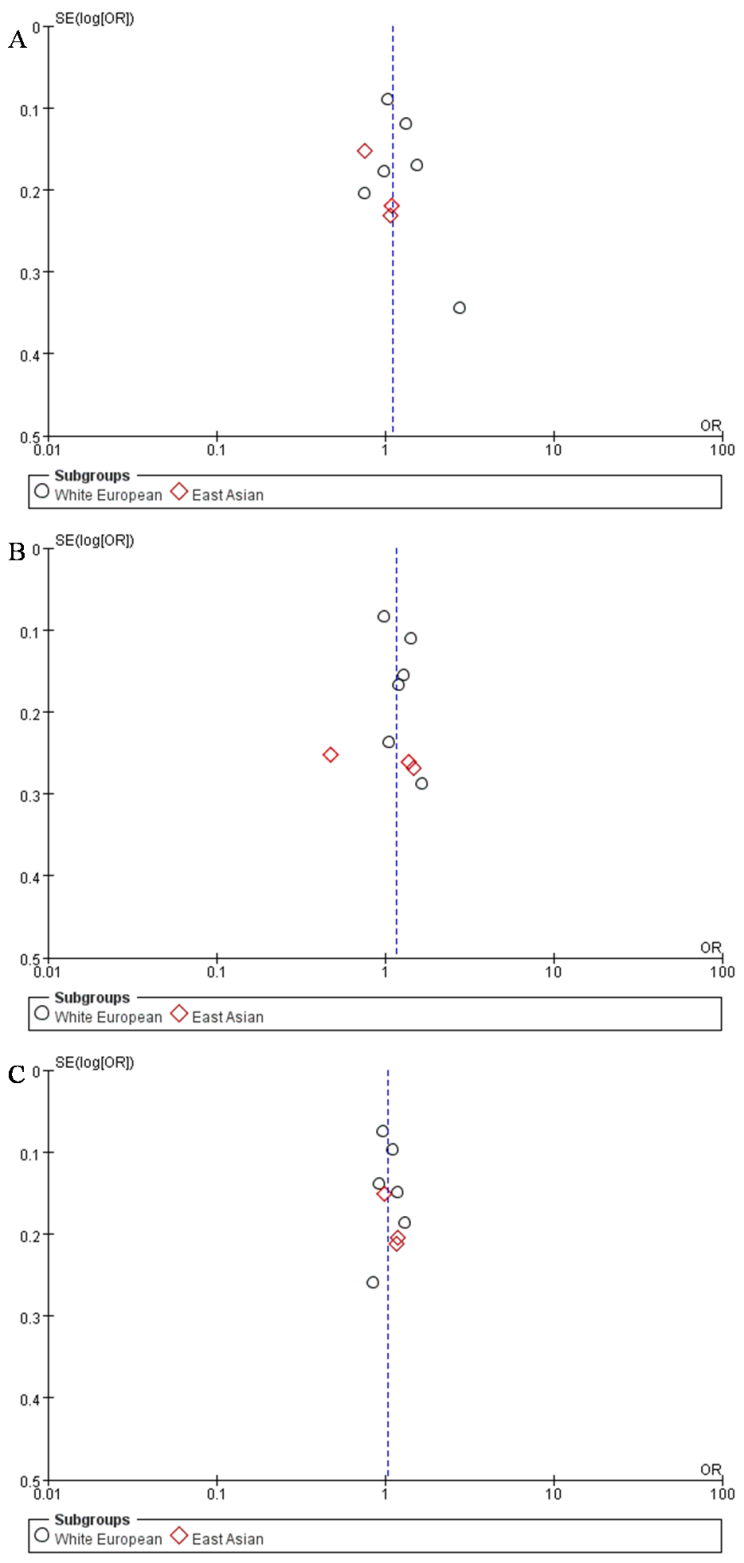
Funnel Plot of different model The shapes of the funnel plots show a slight asymmetry. **A.** Dominant model (GG + TG vs TT); **B.** Recessive model (GG vs TG + TT); **C.** Additive model (GG +TT vs TG).

Funnel plots were used to assess publication bias in different models. The shapes of the funnel plots showed a slight asymmetry (Figure 3). If we include the study who has the same research population, there shouldn't have publication bias.

## DISCUSSION

Our meta-analysis, which included 5008 cases and 3718 controls, assessed the association between the rs6983267 polymorphism and risk of prostate cancer. Our results indicated that the GG genotype of the rs6983267 polymorphism was a risk factor for prostate cancer [OR = 1.21 (95% CI: 1.03, 1.42), P = 0.02) in white European men. However, no association was observed after performing meta-analysis for all of the ethnicities (95% CI: 0.95, 1.41), P = 0.58, especially for Asian people (95% CI: 0.47, 2.04), P = 0.96).

There are some problems which are needed to pay attention to in future. First, according to the criterion of meta-analysis about the inclusion and exclusion of the previous study, other SNPs were not selected in this meta-analysis partly because the lack of sufficient studies which were used to meta-analysis, on the other hand due to the lack of enough data among the total studies. Second, in this study, we included 9 studies that 6 focus on the White European and the rest as the East Asia country. Considering the concentration, we conducted subgroup analysis on the basis of their study population which means we divided 9 studies into White European subgroup and East Asian subgroup, or you can call the later Non-White European subgroup. This process will reduce the heteroplasmy influence of different population and lead to more reliable and refined results. However, the participants included in these studies which were conducted in Europe did not belong to the same country. Third, only 3 studies performed in East Asia countries were included in the meta-analysis, which is not sufficient for the process of meta-analysis in strict criterion. Therefore, more independent case-control studies should be performed in different countries to obtain more comprehensive results. Besides, their study population was so small and different in the later subgroup that we can't give precise result according to the statistical result of the later subgroup analysis.

Overall, though the GG genotype of the rs6983267 polymorphism is not a risk factor of prostate cancer for all of the ethnicities, presence of this genotype in white European men will increase their susceptibility to prostate cancer. Because the results were obtained by sampling statics and because statistical difference is not the same as clinical difference, these results can be used for clinical reference but not for diagnosing prostate cancer. Further detailed studies involving larger number of participants should be performed worldwide to clarify the role of this polymorphism in the risk of prostate cancer. The interplay between rs6983267 and other risk genetic factors about prostate cancer should also be investigated.

## MATERIALS AND METHODS

### Search strategy

A comprehensive literature search for suitable studies published before 2015 was conducted in PubMed, Web of Science and Embase database by using keywords “rs6983267” and “prostate cancer”. Studies that investigated the association between the rs6983267 polymorphism and risk of prostate cancer were included in this meta-analysis. Studies had to be published as a full paper.

### Criterion for study selection

Two independent reviewers screened titles and abstracts to identify relevant studies. Full-text articles of these studies were then read to select eligible studies. Studies were included in the meta-analysis if (1) they were case-control studies, (2) they evaluated the association between the rs6983267 polymorphism and risk of prostate cancer, (3) they provided the number of genotypes in case-control groups for calculating odds ratios (ORs) with 95% confidential intervals (CIs), (4) genotypes of participants in control groups satisfied Hardy–Weinberg equilibrium (HWE) and (5) they were not included in other meta-analyses.

### Data extraction

Following data were extracted from each study included in the meta-analysis by 2 independent reviewers: name of the first author, publication date, country of study participants and number of genotypes in case-control groups. In addition, information on the association between the rs6983267 polymorphism and risk of prostate cancer and *P* value according to the HWE were extracted from the included studies.

### Statistical analysis

The meta-analysis was performed using Cochrane Collaboration Review Manager 5.3. ORs with 95% CIs were calculated to evaluate the association between the rs6983267 polymorphism and risk of prostate cancer. In addition, subgroup analysis was performed according to the race of study participants. *Q* and *I^2^* statistical magnitudes were used to assess heterogeneity. Theoretically speaking, we choose random effect model if there are heterogeneity. Otherwise, we choose fixed effect model. Different studies showed different degrees of heterogeneity. CIs calculated using random effects model were larger than those calculated using fixed effects model. Moreover, results obtained with the random effects model were more conservative. Therefore, the random effects model was chosen. Publication bias was analysed using funnel plots. *Z* test was used to verify the diversity between case and control groups, and the diversity was considered evident at *P* < 0.05.
